# Supplementary material for: Patterns of Intron Gain and Loss in Fungi
Source: PLoS Biol. 2004 Nov 30;2(12):e422. doi: 10.1371/journal.pbio.0020422 (PMC532390; doi:10.1371/journal.pbio.0020422)
Supplement: Table S1 — Also available at http://genes.mit.edu/NielsenEtAl/. (4.3 MB ZIP). [file pbio.0020422.st001.zip › NielsenEtAl/html/749.html]

AN6073.1.NCU03310.1.MG09886.1.FG01119.1


```
 CLUSTAL W (1.82) Multiple Sequence Alignments - Introns Inserted


Sequence 1: NCU03310.1	310 aa
Sequence 2: MG09886.1	303 aa
Sequence 3: FG01119.1	305 aa
Sequence 4: AN6073.1	307 aa
Alignment Length: 311 aa
Number Identitical Residues: 202 aa
Alignment Score (without introns) 8247


MG09886.1 	MSQNPFQEY-MRQLQRIQQRAGGG---GGMPPRGTIGLG---AAAALGAAGIWVVSNSLF
NCU03310.1	MSNNPYQDL-LNKIQQTARSRGGGGFPGGRPPIPPRGLGPALTGFALLGGGAWVLSNSLF
FG01119.1 	MSNNNWQEEAMRRLRQMQQARGGG---GGGPQMP-RAAGGALAGGLLLAGGALFLSNSLF
AN6073.1  	MSRIPKDQW--ERLQLILQSRNRFN--GFRPGGGGGGGIGASAALIVLGLGGWALSNSLF
          	**.   ::   .:::   :  .  .  *  *     .   : :.  : . *   :*****

MG09886.1 	NV~DGGHRAIKYRRISGVSKEIFGEG1THFAIPWFETPIVYDVRAKPRNVSSLTGTKDLQ
NCU03310.1	NV~DGGHRAIKYRRVNGVSKEIYGEG1THLMIPWFETPITYDVRAKPRNVSSLTGTKDLQ
FG01119.1 	NV~DGGQRAIKYQRLTGVSKEIYNEG1THINIPWFETPIVYDVRAKPRNVASLTGTKDLQ
AN6073.1  	NV1DGGHRAIKYSRFGGVKKEIYSEG1THFAIPLIETPIIYDVRAKPRNIASLTGTKDLQ
          	** ***:***** *. **.***:.** **: ** :**** *********::*********

MG09886.1 	MVNITCRVLSRPEVKALPQIYRTLGSDYDERVLPSIVNEVLKSVVAQFNASQLITQRENV
NCU03310.1	MVNITCRVLSRPEVTALPQIYRTLGTDYDERVLPSIVNEVLKSVVAQFNASQLITQREMV
FG01119.1 	MVNITCRVLSRPQIDALPQIYRTLGTDYDERVLPSIVNEVLKSVVAQFNASQLITQRENV
AN6073.1  	MVNITCRVLSRPRVDALPQIYRTLGQDFDERVLPSIVNEVLKSVVAQFNASQLITQRENV
          	************.: ********** *:****************************** *

MG09886.1 	ARLIRENLSRRAALFNIVLDDVSLT0HLAFSPEFTAAVEAKQVAQQEAQRAAFVVDKARQ
NCU03310.1	AKLVRENLAKRAARFNILLDDVSLT0HLAFSPEFTAAVEAKQVAQQEAQRAAFIVDKARQ
FG01119.1 	ARLVRENLARRAARFNILLDDVSLT0HLAFSPEFTAAVEAKQVAQQEAQRAAFVVDKARQ
AN6073.1  	ARLVRDNLARRAARFNIALDDVSLT0HLTFSPEFTAAVEAKQVAQQEAQRAAFLVDKARQ
          	*:*:*:**::*** *** ******* **:************************:******

MG09886.1 	EKQAMVVKAQGEARSAELIGEAIKKSKSYVELKKLENARAIAQTLQEAGGRNRLLLDAEG
NCU03310.1	EKQAMVVKAQGEARSAELIGEAIKKSKSYVELKKLENARAIANIIQEAGGKNRLLLDSEG
FG01119.1 	EKQAMVVKAQGEARSAELIGEAIKKNKAYLELKKIENARLIAAQLQEAGSKNRLMLDSEG
AN6073.1  	EKQAFIVRAQGEARSAELIGDAIKKSKSYIELRRIENARHIAQIIQENGGRNKLYLDSQG
          	****::*:************:****.*:*:**:::**** **  :** *.:*:* **::*

MG09886.1 	LGLNVFEKTDRKD-
NCU03310.1	LGLNVFEDRRSKDN
FG01119.1 	LGLNVFDKNDKS--
AN6073.1  	LGLNVNAGADGESK
          	*****      ...
```
